# A potential mechanism of the onset of immune-related pneumonitis triggered by anti-PD-1 treatment in a patient with advanced adenocarcinoma lung cancer: case report

**DOI:** 10.1186/s12890-021-01649-6

**Published:** 2021-09-14

**Authors:** Yu Feng, Cuncun Chen, Liming Zhao, Xuyou Zhu, Xiaoping Zhu, Qiang Li

**Affiliations:** 1 Department of Respiratory Medicine, Shanghai East Hospital, Affiliated to Tongji University, Shanghai, 200120 China; 2 Department of Clinical Laboratory, Shanghai East Hospital, Affiliated to Tongji University, Shanghai, 200120 China; 3 Department of Pathology, Tongji Hospital, Affiliated to Tongji University, Shanghai, 200438 China

**Keywords:** Immune-related pneumonitis, Immune reconstitution inflammatory syndrome, Anti-PD1, Case report

## Abstract

**Background:**

In recent years, the application of immunotherapy combined with chemotherapy in the first-line lung cancer has showed significant benefit in improving long-term survival. Immunotherapy also has risks of immune-related pneumonitis (IRP) after long-term treatment. Despite the treatment strategy of the IRP has been very clear. However, the mechanism is unclear.

**Case presentation:**

A 73-year-old male patient was diagnosed with left lung adenocarcinoma IVa, EGFR, ALK, ROS1 negative. The patient received anti-PD1 antibody combined with pemetrexed and cisplatin. After 5 cycles of treatment, partial response was obtained. Subsequently, the patient continued the treatment of anti-PD1 antibody combined with pemetrexed. Before the 7th cycle, the CT found a new lesion in the basal segment of the right lower lobe. It was diagnosed with IRP and pneumocystis jirovecii. The patient did not give trimethoprim–sulphamethoxazole (TMP–SMX) and corticosteroids, symptoms and radiological lesions had improved. We describe the report of immune-related pneumonitis trigged by anti PD-1 and monitored the dynamic changes of CD^4+^, CD^8+^ T lymphocytes, MDSC and Treg cells in the bilateral bronchoalveolar alveolar lavage fluid. From the point of view of immune cells, the mechanism of immune reconstitution inflammatory syndrome is confirmed. Based on the current case report and literature, this study proposes a potential mechanism of the onset.

**Conclusion:**

Immune reconstitution inflammatory syndrome may be potential mechanism of IRP. This study may improve our understanding of the pathogenesis underlying IRP. We believe the detection and dynamic monitoring CD4^+^, CD8^+^ T lymphocytes, MDSC and Treg cells can provide more accurate procedures.

## Background

Recently, immunotherapy of lung cancer has been widely used, immune-related pneumonitis (IRP) are gradually known. In addition, with regard to reports of infections (tuberculosis, fungi) in the lungs of patients with non-immune deficiency after immunotherapy, some scholars believe that this phenomenon is attributed to the immune reconstitution inflammatory syndrome (IRIS) activated by immunotherapy. Here, we found a patient with IRP and pneumocystis jirovecii infection occurred after anti-PD1 antibody treatment. The mechanism of IRP has been explored and identified for this patient.

## Case presentation

**Fig. 1:**
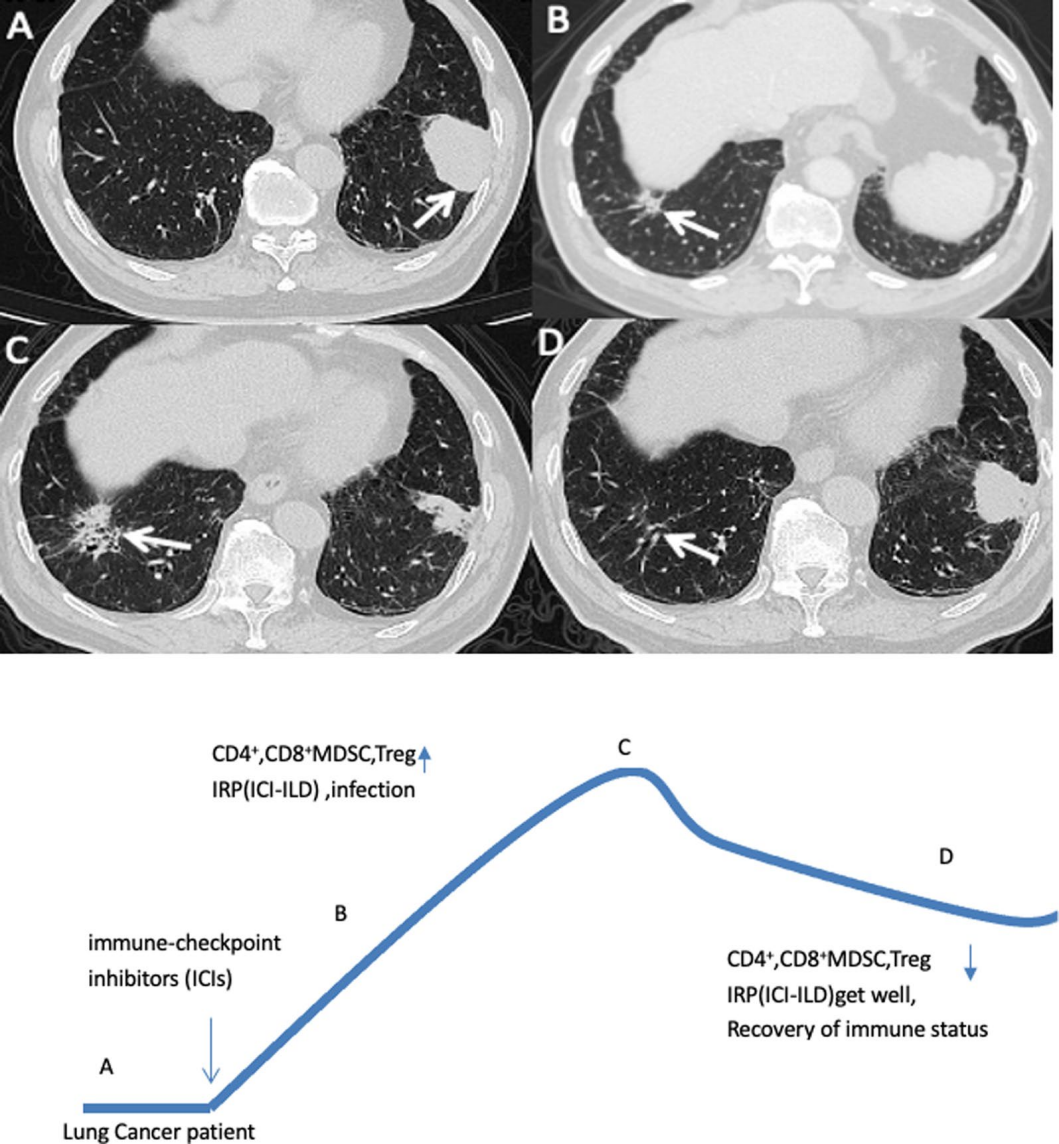
Computed tomography (CT) images during the clinical course of the patient. **A** lung adenocarcinoma of the left lower lobe (arrow), **B** new lesions of the right lower lobe (arrow), **C** progress of new lesions of the right lower lobe (arrow), partial remission of the lung adenocarcinoma of the left lower lobe, **D** regression of lesion of the right lower lobe (arrow), **E** Mechanism of immune-related pneumonitis caused by immune reconstitution inflammatory syndrome

A 73-year-old male patient was diagnosed with left lung adenocarcinoma T3N3M1b stage IVa, EGFR, ALK, ROS1 negative, PDL1 1–49%. The patient received anti-PD1 antibody combined with pemetrexed and cisplatin. After 5 cycles of treatment, partial response was obtained. The patient did not experience neutropenia and lymphopenia during chemotherapy, but showed anemia. Subsequently, the patient continued the treatment of anti-PD1 antibody combined with pemetrexed. Before the 7th cycle, the CT found a new lesion in the basal segment of the right lower lobe (Fig. 1A, B). The hematology test suggested that white blood cells 5.09*10^9^/L (3.5–9.5*10^9^/L), neutrophil count 1.90*10^9^/L (1.8–6.30*10^9^/L), lymphocyte count 1.66*10^9^/L (1.10–3.20*10^9^/L), hemoglobin 82 g/L (130–175 g/L), blood platelet count 297*10^9^/L (125–350*10^9^/L), The new lesion was considered as treatment related adverse event rather than disease progression. So we recommended to postpone the treatment to furtherly identify the lesion to be immune-related pneumonitis (grade 1) or lung infection. However, the patient claimed that he had no fever, no chills, no chest tightness, no cough and expectoration, shortness of breath and required continuation of immunotherapy. Thus, the 7th treatment of anti-PD1 antibody combined with pemetrexed was administered, we gave the patient levofloxacin tablets empirically orally for 2 weeks after completion of treatment. After 2 weeks, the fever suddenly occurred with body temperature of 38.3 °C. the patient also represented the chest tightness, shortness of breath, difficulty breathing, fatigue, and scattered red spots were seen all over the body. The hematology test suggested that white blood cells 5.90*10^9^/L (3.5–9.5*10^9^/L), neutrophil count 3.99*10^9^/L (1.8–6.30*10^9^/L), lymphocyte count 0.83*10^9^/L (1.10–3.20*10^9^/L), hemoglobin 73 g/L (130–175 g/L), blood platelet count 112*10^9^/L (125–350*10^9^/L), and neutrophils 67.6% (within the normal range), peripheral blood CD4^+^ 43%(25.8–41.6%), CD8^+^10.7%(18.1–29.6%) and a negative HIV test. The chest CT revealed the right lower lung base segmental lesions increased compared with the previous one (Fig. 1C).

**Fig. 2:**
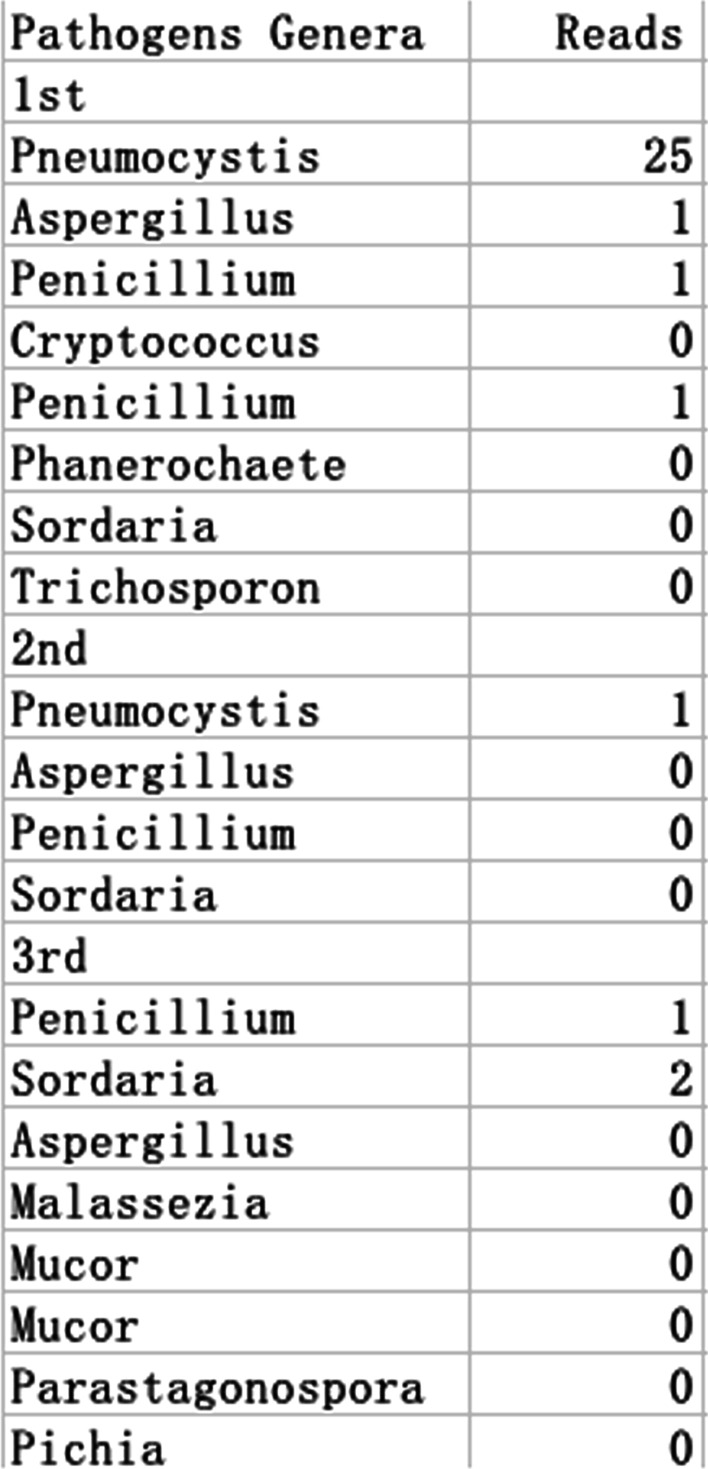
BALF next-generation sequencing test results, the 1st test showed 25 reads of Pneumocystis jirovecii, the 2nd test showed 1 read, and the 3rd test was negative

**Fig. 3:**
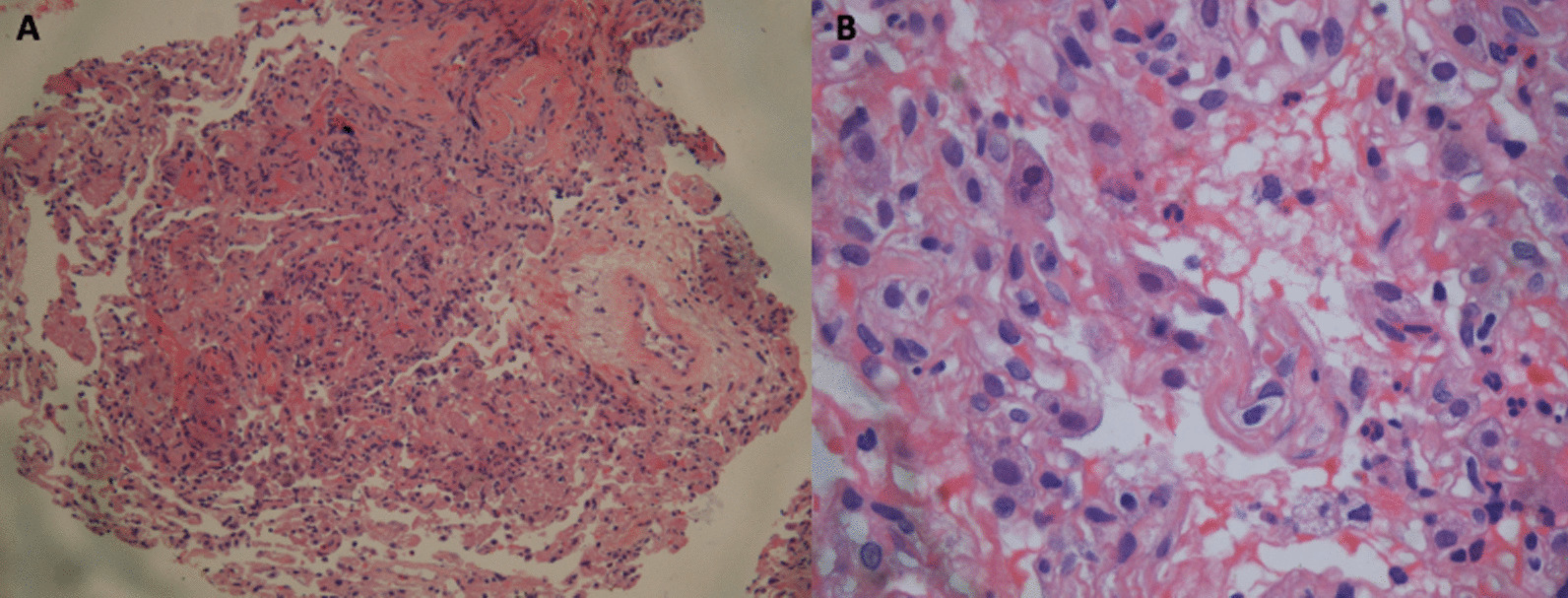
Histopathological findings of examination of the right lower lobe biopsy specimens. Interstitial fibrous tissue hyperplasia, fibroblast proliferation in alveolar cavity, alveolar inflammation (hematoxylin and eosin). **A** Original magnification, × 100; **B** original magnification, × 400)

**Fig. 4:**
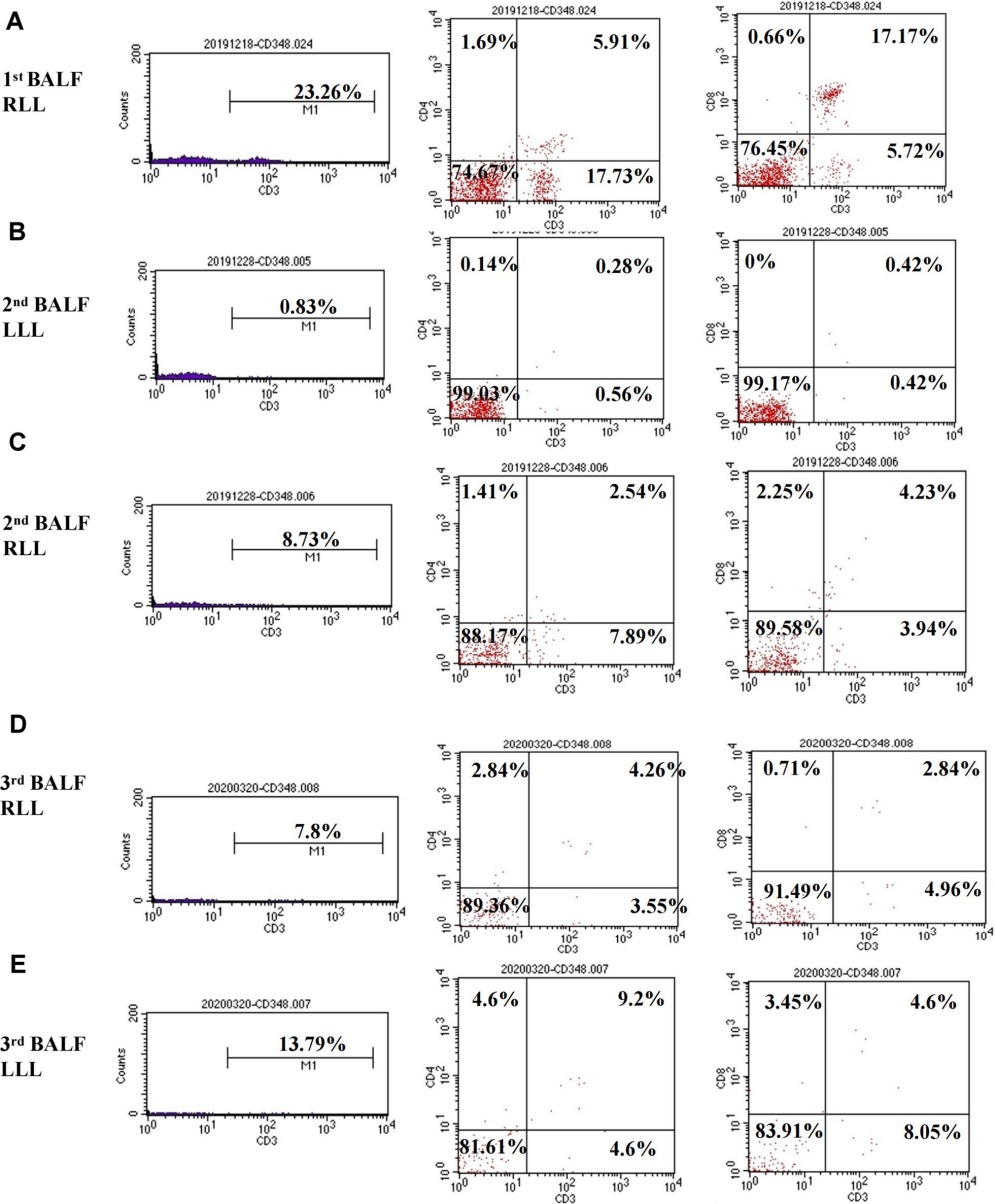
Expression of CD^3+^, CD^4+^ and CD^8+^ T cells s in BALF. **A** The expression of CD^3+^, CD^4+^ and CD^8+^ T cells in the 1st BALF of right lower lobe (RLL). **B** The expression of CD^3+^, CD^4+^ and CD^8+^ T cells in 2nd BALF the left lower lobe (LLL). **C** The expression of CD^3+^, CD^4+^ and CD^8+^ T cells in the 2nd BALF of right lower lobe (RLL). **D** The expression of CD^3+^, CD^4+^ and CD^8+^ T cells in the 3rd BALF of left lower lobe (LLL). **E** The expression of CD^3+^, CD^4+^ and CD^8+^ T cells in the 3rd BALF of right lower lobe (RLL)

**Fig. 5:**
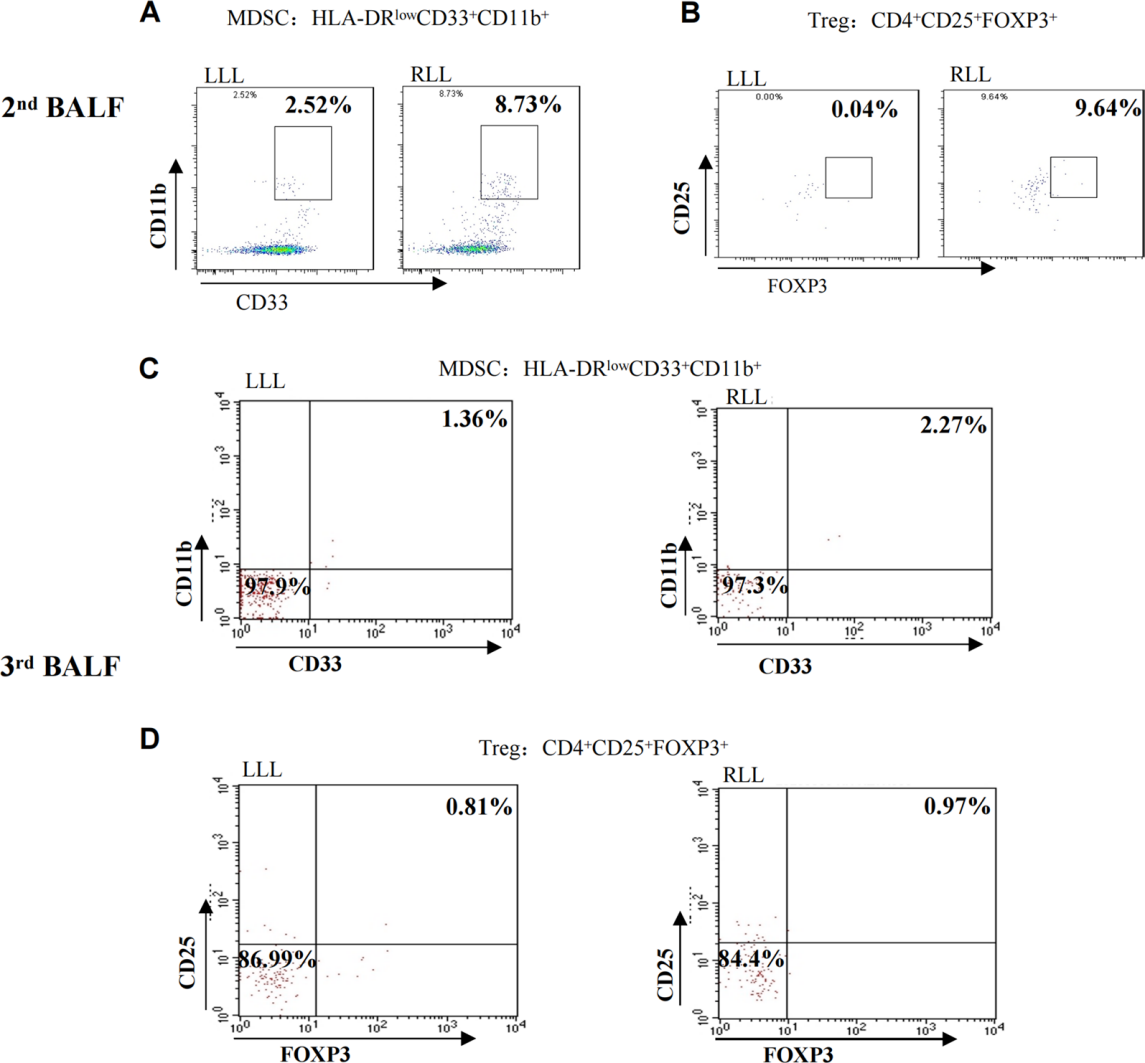
Expression of MDSC (HLA-DRlowCD33+CD11b+), Treg (CD4+CD25+FOXP3+) in bilateral BALF. **A** The expression of MDSC in the 2nd BALF of left lower lobe (LLL) and right lower lobe (RLL). **B** The expression of Treg in the 2nd BALF of left lower lobe (LLL) and right lower lobe (RLL). **C** The expression of MDSC in the 3rd BALF of left lower lobe (LLL) and right lower lobe (RLL). **D** The expression of Treg in the 3rd BALF of left lower lobe (LLL) and right lower lobe (RLL)

We suspected the patient had developed immune-related pneumonitis (grade 2) and suspended the next cycle of immunotherapy, performed corticosteroid therapy and bronchoscopy. However, this elderly patient had osteoporosis and was very resistant to the use of glucocorticoids. In the first bronchoscopy, bronchoalveolar lavage fluid (BALF) was performed on the right lower lobe lesion, the detection of the pathogenic microorganism next-generation sequencing (NGS) of BALF revealed that pneumocystis jirovecii 25 reads indicating pneumocystis jirovecii infection (Fig. 2). BALF cell count classification showed 90% lymphocytes, 2% neutrophils, CD3^+^ (23.26%), CD4^+^ (5.91%), CD8^+^ T cell (17.17%) (Fig. 4A). We consider that the patient was non-HIV, non-corticosteroid immunodeficiency, and CD4 count was not less than 200 cells, only used antipyretic drugs but did not give trimethoprim–sulphamethoxazole (TMP–SMX) and corticosteroids. After ten days of supportive care and three weeks after discontinuation of immunotherapy, the patient's fever, fatigue, chest tightness, shortness of breath, dyspnea, and rash improved significantly. The second bronchoscopy using EBUS-GS-TBLB, was done with biopsy of the right lung lesions. In the second BALF, NGS suggested that pneumocystis jirovecii decreased to 1 read. BALF Jimussa staining showed no cysts and trophozoites. The pathology of the right lower lobe biopsy showed alveolar inflammation and no malignant tumor cells (Fig. 3). In this time, we collected BALF at bilateral lung lesions with detection of CD4^+^ CD8^+^ T cells. The results showed that CD3^+^ (0.83%), CD4^+^ (0.28%), and CD8^+^ (0.42%) T cells in BALF of the left lower lobe (Fig. 4B), CD3^+^ (8.73%), CD4^+^ (2.54%), and CD8^+^ (4.23%) T cells in BALF of the right lower lobe, respectively (Fig. 4C), Additionally, we tested MDSC and Treg cells, the results suggested that there was a difference between bilateral BALF, with MDSC 2.52% in left side and 8.73% in right side (Fig. 5A), Treg cells were 0.04% and 9.64% respectively (Fig. 5B).

The CT (2 months after discontinuation of immunotherapy) showed that the right lower lobe lesion was completely absorbed (Fig. 1D). Thus we conducted the third bronchoscopy, the NGS test was negative for pneumocystis jirovecii, and the BALF of the bilateral lung lesions were again detected, CD3^+^ (7.8%), CD4^+^ (4.26%), and CD8^+^ (2.84%) T cells in BALF of the right lower lobe (Fig. 4D), CD3^+^ (13.79%), CD4^+^ (9.2%), and CD8^+^ (4.6%) T cells in BALF of the left lower lobe respectively (Fig. 4E). At this time, the percentage of MDSC and Treg cells were very similar in left and right side BALF. MDSC were 1.36% and 2.27% (Fig. 5C), also Treg cell 0.81% and 0.97% respectively (Fig. 5D).

## Discussion and conclusion

With the widespread use of immunotherapy in lung cancer, the main concern is diagnosis and management of immune-related adverse event (irAEs). The incidence of irAEs is approximately 66% present including fatigue, itchy skin, coughing, fever and dyspnea, etc [1], among which the incidence of IRP was between 1.4%and 5.8% in NSCLC [2], imaging presentation of organizing pneumonitis, nonspecific interstitial pneumonitis, hypersensitivity pneumonitis [2, 3]. The pathology of IRP is interstitial pneumonia, and different types of interstitial pneumonia have different mechanisms, is very complicated.

This patient manifested fever, nausea, fatigue, chest tightness, shortness of breath, dyspnea, and rash all over the body. In order to rule out infectious diseases, we performed blood test and bronchoscopy, pathogen detection in BALF (including NGS technology) and pathological examination). In the first examination of bronchoscopy, the classification of BALF (right lesion) cells was mainly consist of lymphocytes, these are consistent with the published report [4, 5]. The patient's blood test suggested that white blood cells and neutrophils are within the normal range. Based on the overall evidence, the patient was diagnosed with IRP, grade 2. Current guidelines recommend a dose of 1 mg/kg/day of prednisone for grade 2 and 2-4 mg/kg/day for grade 3–4. We also believe that the patient should receive corticosteroid treatment immediately, but unfortunately the patient was worried about the side effects and refused. When analyzing the second BALF, we found that there was a difference CD4^+^ and CD8^+^ T cells in BALF between the primary tumors (left side) and IRP (right side). After immunotherapy, the counts of CD4^+^ and CD8^+^ T cells in BALF at the tumor lesion (left side) were lower than the CD4^+^ and CD8^+^ T cells in the right side, the same trend was seen in MDSC and Treg cells [6]. On the other hand, we detected pneumocystis jirovecii twice in BALF (on the right), is likely to be secondary to immunotherapy. We consider that the non-HIV patient had no long-term history of taking high-dose corticoids and immunosuppressive drugs, the CD4 count was > 200 cells/μL, the evidence for TMP–SMX prophylaxis is not very sufficient. PJP prophylaxis is depends on the immune status of the patient and the underlying immunocompromising disease [7]. Although dexamethasone was used in pemetrexed, the dose of dexamethasone (4 mg, orally twice per day. Taken the day before, day of, and day after pemetrexed administration) in our protocol was in accordance with the standards of the international clinical trial protocol (NCT02578680-Keynote189, NCT03215706-CheckMate 9LA) with similar study design investigating anti-PD1 combined with chemotherapy in first line metastatic NSCLC. There is currently no report on the prevention of allergic doses of dexamethasone that can cause immunodeficiency. In contrast, currently most of the reported cases of PJP (pneumocystis jirovecii pneumonia) after immunotherapy are due to the usage of glucocorticoid or TNF-α inhibitors in the emergence of grade 3–4 irAEs [8]. This patient had not been given the treatment of corticosteroids or TNF-α inhibitors according the irAEs management guidelines. There have been cases reported that tuberculosis and fungal infection have occurred patients with non-immune deficiency secondary to immunotherapy [9–11], considering the hypersensitivity of drug agents induced by the immune checkpoint inhibitors [12], thus, immune reconstitution inflammatory syndrome (IRIS) is activated. We believe that this patient also experienced non-HIV IRIS secondary to immunotherapy based on the draft diagnosis criteria of non-HIV IRIS proposed by Sueki et al. [13].^.^ HIV IRIS generally refers to the symptoms of opportunistic infections worsening after AIDS patients' immune function recovers after HAART treatment. Non-HIV IRIS can have two manifestations, one is that previously acquired opportunistic infection exacerbation, and the other it can be a sudden increase in an invisible infection that has not been infected before, such as this case and the reported [9]. Immune checkpoint inhibitor is one of risk factors for IRIS occurred [13, 14]. The disorder was caused by an antigen (checkpoint inhibitor) and associated with recovery from an immunocompromised condition due to advanced cancer and/or checkpoint inhibitor which is their central pathogenesis. We found that there was a significant difference in the immune environment between the tumor lesion and the IRP in the bilateral lungs. In the BALF with IRP and opportunistic infection on the right side, CD^4+^, CD^8+^, MDSC, Treg were found to rise first and then fall back, indicating that the immunosuppressive state has become activated and the immune balance state is restored (Fig. 1E). The significant increase in CD^4+^ and CD^8+^ T cells led to IRP, there are studies on the range (0.2–0.8) CD4/CD8 ratio of IRP, the normal range (0.8–2) that can assess the diagnosis of IRP [15]. The CD4/CD8 ratio of the three BALFs in our study were 0.3, 0.6, 1.5, respectively. Treg plays an important role in immune regulation. On the one hand, Treg is generally elevated during irAEs. On other hand, Treg also can inhibit anti-Pneumocystis CD^4+^ T cells during IRIS [16]. It is implying that there is a correlation between the occurrence of IRP and IRIS.

In addition, according to the NGS of the pathogenic microorganism, the reads of pneumocystis jirovecii after last time of immunotherapy was higher, this means pneumocystis-related IRIS was aggravated (Fig. 1B, C) and CD4, Treg, and MDSC were significantly increased. With Treg from 9.64 to 0.97, MDSC from 8.73 to 2.27, the reads decreased to normal, and the immune system was balanced finally (Fig. 1C, D). We speculate that while IRIS has taken place in the Fig. 1B, then used the PD-1 again, IRIS aggravated thereby presenting symptoms get worse and opportunistic infections. PJP may be self-limiting in some cases [17]. The IRIS of lung cancer may be different from that of HIV patients. PJP will recover with the immune balance. This argument needs further research in the future.

The mainstay of management of IRP (G2-G4) is corticosteroid therapy, but due to the individual condition of patient, the corticosteroid was not administered. The results of this study highlight the need for future research.

In conclusion, this patient should have triggered IRIS after using immune checkpoint inhibitors. Immune reconstitution inflammatory syndrome may be a potential mechanism of IRP. This study may improve our understanding of the pathogenesis underlying IRP.

## Data Availability

The data that support the findings of this case are available from the corresponding author upon reasonable request.

## Abbreviations

irAEs: Immune-related adverse events
IRP: Immune-related pneumonitis
IRIS: Immune reconstitution inflammatory syndrome
PD-1: Programmed death-1
BALF: Bronchoalveolar alveolar lavage fluid
PJP: Pneumocystis jirovecii pneumonia
MDSC: Myeloid-derived suppressor cells
NSCLC: Non small cell lung cancer
HAART: Highly active antiretroviral therapy
